# Distribution of blood pressure in school going children in rural area of Wardha district, Maharashatra, India

**DOI:** 10.4103/0974-2069.43874

**Published:** 2008

**Authors:** Amar Taksande, Pushpa Chaturvedi, Krishna Vilhekar, Manish Jain

**Affiliations:** Department of Pediatrics, Mahatma Gandhi Institute of Medical Sciences, Sewagram, Wardha, India

**Keywords:** Blood pressure, children, hypertension, prevalence study

## Abstract

**Objectives:**

To study the blood pressure of school going children in a rural area and its relationship with the anthropometric indices.

**Methods:**

A prospective, cross-sectional study was carried out from November 2006 to December 2007 in the school going children between the ages of 6–17 years from eight different schools in the rural areas of Wardha district. The height, weight, systolic blood pressure (SBP), and diastolic blood pressure (DBP) were recorded in both sexes followed by complete clinical examination with special emphasis on cardiovascular system. Hypertension (HT) was defined as SBP or DBP exceeding the 95th percentile for age, gender, and height on at least three separate occasions, 1–3 weeks apart. SPSS software was used to analyze the data. Coefficient correlation tests were employed to assess the relation between BP and anthropometric variables.

**Results:**

Of 2643 school children, 1227 were boys and 1416 girls with a male to female ratio of 1:1.16. In boys, SBP and DBP increased with age except a marginal decline in SBP at the age of 17 years (−0.09) and decrease in the DBP (−1.29) at 16 years of age. In girls, SBP and DBP also increased with age except at 11 years, wherein there was a mild decrease in SBP (−0.09) as well as the DBP (−0.24). Correlation coefficient analysis showed highly significant positive correlation of height with SBP and DBP. There was a significant correlation of SBP and DBP with the weight, and body mass index (BMI). The prevalence of HT was 5.75% (i.e., 3.25% for systolic HT and 2.49% for diastolic HT).

**Conclusion:**

We recommend that there is a need for checking BP to detect HT in children, so that remedial measures can be initiated as early as possible.

## INTRODUCTION

Hypertension (HT) is a major health problem in developed and developing countries. Around one billion adult world population was found to have HT in the year 2000 and this is expected to increase to 1.56 billion by 2025.[1,2] The incorporation of blood pressure (BP) measurement into routine pediatric examination has lead to the discovery of significant number of children with asymptomatic HT.[3] Since the risk factors for the development of HT start in childhood, pediatricians should be encouraged to include routine BP measurement in children.[4] Systemic HT has an estimated population prevalence of 1–2%[5] in the developed countries and 5–10% in developing countries like India.[6] The risk factors for HT include obesity, family history of HT, change in dietary habits, decreased physical activity, and increasing stress.[7] Although the overall prevalence of HT is lower in children, studies suggest that it tends to develop during the first two decades of life.[8] Luepker *et al*,[9] reported that BP normally increases with growth and development. Children with higher BP tend to maintain those levels of BP in adulthood.[10] HT is a risk factor for ischemic heart disease. Thus, it is necessary to detect and effectively treat HT during childhood and adolescence. This study was planned to determine the prevalence of HT in school children and to correlate it to anthropometric parameters.

## METHODS

This prospective cross-sectional study was conducted as a part of the school health examination survey involving children and adolescents attending rural middle secondary schools in Wardha district. The schools were randomly selected after prior permission and consent from the concerned authorities. The importance of the study was explained to the school management, staff, and teachers. A total of 2643 school children in the age range of 6–17 years were examined from eight schools, between November 2006 and May 2007. The absentees could not be examined. Age, gender, religion, address, and complaints were recorded. The age was determined from the birth date of the school registration record. All children were clinically examined with special emphasis on evaluation of cardiovascular system. Anthropometric indices were recorded as per the recommendations.[11] The weight was recorded to the nearest 0.1 kg by weighing scale and the height was noted to the nearest 0.5 cm using a measuring scale. BMI was calculated by the formula: BMI = weight (kg)/height (m)^2^.

The BP measurement and clinical examination of the children was done in the afternoon during the school hours. BP was measured using standardized sphygmomanometers with appropriate size cuff, covering two-thirds of the arm. The BP was measured with the child in a sitting position, with the arm at the level of the heart, and after a five-minute rest. The cuff was inflated to a level at which the distal arterial pulse was not palpable. It was then deflated at a rate of 2–3 mm Hg per second. SBP was recorded on hearing the first sound (phase I), while DBP was taken on complete disappearance of Korotkoff sounds (phase V). Prehypertension is defined as SBP or DSP between the 90th and 95th percentile. Adolescents having blood pressure >120/80 mm Hg, but below the 95th percentile are also included in this category. HT is defined as SBP or DBP exceeding the 95th percentile for age, gender, and height on at least three separate occasions, 1–3 weeks apart.[6] If the SBP was higher than 120 mm Hg and the DBP higher than 80 mm Hg, two additional readings were obtained and crosschecked by another consultant. The lowest of the three readings was recorded.[8] All the readings were made by the same observer to avoid inter-observer variation. Statistical analysis was carried out using the SPSS 10 version. The mean, standard deviation, and annual increase were calculated. Coefficient of correlation was calculated to assess the relation between BP and anthropometric variables. A value of *P* < 0.05 was considered as statistically significant.

## RESULTS

Of 2643 school children, 1416 (53.57%) were girls and 1227 (46.43%) boys with a female to male ratio of 1.16:1. The height and weight of boys were significantly more than that of girls of the same age group. The mean, standard deviation, and annual rate of increment of SBP and DBP in boys and girls is summarized in table 1. In boys, SBP and DBP increased with age except at 17 years, wherein there was a marginal decline in SBP (−0.09), and at 16 years, there was a −1.29 decrease in DBP. In girls, SBP and DBP also increased with age except at 11 years when there was a fall in SBP (−0.09) as well as the DBP (−0.24). The mean SBP and DBP did not show statistically significant difference between the two sexes. Among girls, SBP showed a significant rise between 11 and 12 years of age, while in boys this increase was seen between 9 and 10 years. This trend was not evident in DBP.

**Table 1:** Mean, standard deviation, and increments in systolic blood pressure and diastolic blood pressure among boys and girls at different ages in the ascending order

Age (years)	Sex (M/F) and number (n)	SBP (Mean ± SD)	Increment	DBP (Mean ± SD)	Increment
6	M (40)	91.08 ± 4.34	---	61.90 ± 3.46	---
	F (47)	89.87 ± 4.49	---	61.70 ± 3.31	---
7	M (32)	95.57 ± 7.99	4.49	64.43 ± 3.39	2.53
	F (41)	93.90 ± 6.72	4.03	62.02 ± 3.66	0.32
8	M (60)	98.16 ± 8.78	2.59	66 ± 4.31	1.57
	F (70)	96.86 ± 7.13	2.96	63.86 ± 3.64	1.84
9	M (94)	99.62 ± 7.55	1.46	67.75 ± 4.78	1.75
	F (114)	97.80 ± 7.51	0.94	65.86 ± 6.57	2
10	M (42)	102.64 ± 7.88	3.02	68.59 ± 5.08	0.84
	F (54)	100.72 ± 7.27	2.92	67.83 ± 6.01	1.97
11	M (79)	105.63 ± 12.12	2.99	70.89 ± 8.05	2.3
	F (130)	100.63 ± 9.82	−0.09	67.59 ± 8.90	−0.24
12	M (133)	108.36 ± 11.08	2.73	71.83 ± 7.30	0.94
	F (187)	105.71 ± 12.78	5.08	70.20 ± 8.56	2.61
13	M (198)	110.57 ± 11.70	2.21	72.88 ± 8.60	1.05
	F (252)	108.19 ± 11.30	2.48	71.29 ± 8.23	1.09
14	M (224)	112.75 ± 11.86	2.18	73.53 ± 9.44	0.65
	F (215)	110.16 ± 10.84	1.97	72.4 ± 9.10	1.11
15	M (175)	113.49 ± 11.57	0.74	75.11 ± 8.30	1.58
	F (205)	111.85 ± 11.13	1.69	73.67 ± 7.80	1.27
16	M (81)	115.11 ± 12.07	1.62	73.82 ± 9.25	−1.29
	F (85)	112.44 ± 10.42	0.59	73.95 ± 8.73	0.28
17	M (70)	115.02 ± 15.38	−0.09	75.12 ± 9.64	1.3
	F (16)	113.75 ± 8.54	1.31	74.56 ± 7.13	0.61

SBP: Systolic blood pressure; DBP: Diastolic blood pressure

**Figure 1:**
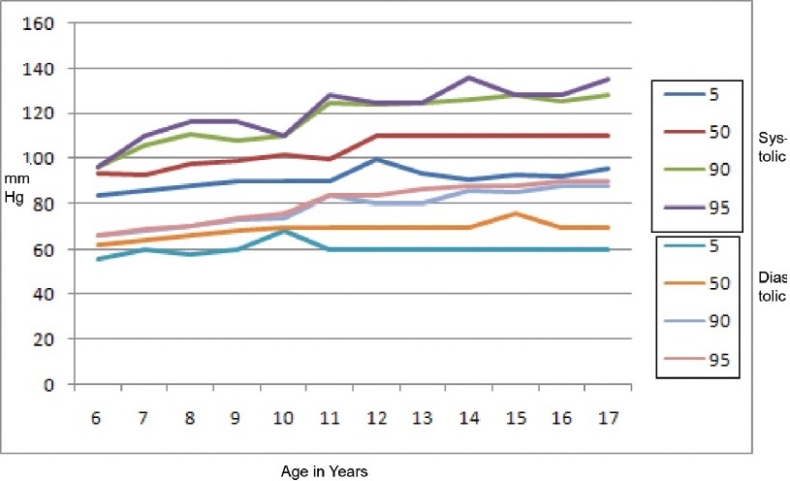
Systolic and diastolic blood pressure percentile for boys

**Figure 2:**
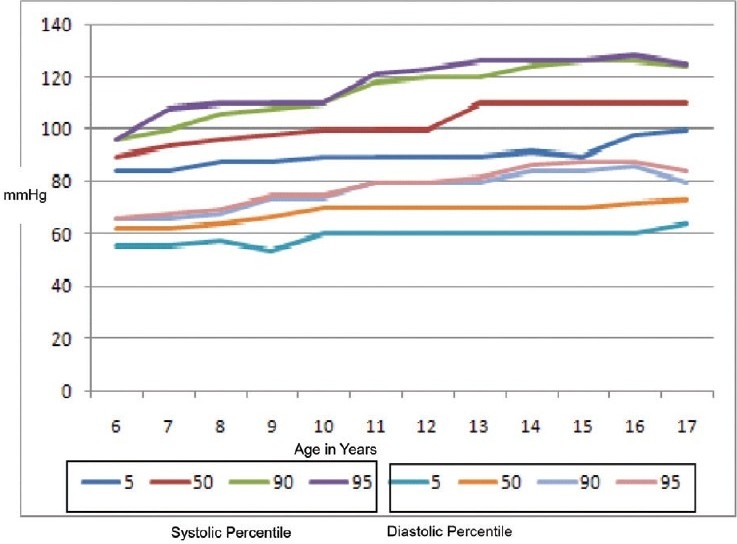
Systolic and diastolic blood pressure percentile for girls

The mean and SD of weight, height, and BMI (anthropometric indices) with respect to age and gender have been tabulated [Table 2]. The distribution of the SBP and DBP by percentiles in various age groups is presented in tables 3 and 4. The 5th, 90th, and 95th percentile curves of the SBP and DBP for both sexes are shown in figures 1 and 2. The SBP and DBP in both girls and boys showed a positive correlation (*P* < 0.001) with age, height, weight, and BMI [Table 5]. The prevalence of HT was 5.75% (*n* = 152). Systolic HT was seen in 3.25% (*n* = 86), while 2.49% (*n* = 66) had DHT. The prevalence was not significantly different among boys and girls.

**Table 2:** Anthropometric indices among boys and girls in different age groups

Age (years)	Sex (M/F) and number (n)	Weight (Mean ± SD)	Height (Mean ± SD)	BMI (Mean ± SD)
6	M (40)	20.9 ± 2.9	117.17 ± 3.04	15.26 ± 2.35
	F (47)	20.46 ± 3.84	116.55 ± 2.94	15.08 ± 2.83
7	M (32)	22.46 ± 2.32	117.84 ± 3.01	16.20 ± 1.80
	F (41)	21.46 ± 2.3	117.07 ± 3.6	15.71 ± 2.03
8	M (60)	24.96 ± 3.93	119.80 ± 3.9	17.51 ± 3.44
	F (70)	23.32 ± 3.4	117.97 ± 4.1	16.84 ± 2.85
9	M (94)	25.20 ± 3.45	121.44 ± 5.72	17.11 ± 2.31
	F (114)	24.69 ± 4.51	120.78 ± 6.85	17.12 ± 3.90
10	M (42)	27.26 ± 4.57	125.16 ± 4.36	17.43 ± 2.95
	F (54)	25.31 ± 5.26	123.94 ± 5.57	16.57 ± 3.76
11	M (79)	29.27 ± 5.41	127.54 ± 7.07	18.07 ± 3.53
	F (130)	26.51 ± 5.66	125.00 ± 6.84	17.03 ± 3.72
12	M (133)	32.62 ± 5.90	132.70 ± 8.07	18.60 ± 3.42
	F (187)	28.88 ± 5.98	130.01 ± 8.05	17.19 ± 3.83
13	M (198)	34.36 ± 7.40	137.32 ± 11.7	18.53 ± 4.86
	F (252)	33.24 ± 6.84	134.81 ± 11.56	18.71 ± 5.22
14	M (224)	36.07 ± 8.95	141.24 ± 11.05	18.28 ± 4.98
	F (215)	34.94 ± 6.63	138.93 ± 7.25	18.20 ± 3.72
15	M (175)	39.29 ± 7.07	145.57 ± 10.48	18.79 ± 4.26
	F (205)	37.04 ± 5.80	141.93 ± 8.54	18.57 ± 3.63
16	M (81)	41.95 ± 7.69	149.58 ± 10.06	18.96 ± 4.30
	F (85)	39.48 ± 6.55	145.44 ± 8.56	18.81 ± 3.62
17	M (70)	46.02 ± 5.90	155.88 ± 8.41	19.04 ± 2.87
	F (16)	41.81 ± 5.94	148.75 ± 6.14	18.91 ± 2.56

BMI: Body mass index

**Table 3:** Distribution of systolic blood pressure by age

Age	Number	Mean (SD)	3	5	10	25	50	75	90	95
6	87	91.48 ± 4.77	82	84	84	88	92	96	98	98
7	73	94.54 ± 7.28	84	84	88	90	94	98	105	108
8	130	97.46 ± 7.76	84	88	90	92	98	100	106	110
9	207	98.62 ± 7.58	86	88	90	92	98	103	108	110
10	96	101.56 ± 7.53	89	90	90	98	100	107	110	116
11	209	103.80 ± 12.16	90	90	90	98	100	110	120	120
12	320	107.05 ± 12.55	90	90	96	100	100	110	120	127
13	450	109.73 ± 11.69	90	90	99	100	110	120	124	130
14	439	111.71 ± 11.76	90	90	100	100	110	120	128	130
15	380	113 ± 11.86	90	90	100	106	110	120	130	130
16	166	114.20 ± 11.81	96	100	100	106	110	120	130	130
17	86	114.95 ± 14.47	96	100	100	110	113	120	130	130

**Table 4:** Distribution of diastolic blood pressure by age

Age	Number	Mean (SD)	3	5	10	25	50	75	90	95
6	87	62.06 ± 3.66	56	56	57	60	62	64	66	68
7	73	63.20 ± 3.73	56	56	58	60	64	66	68	68
8	130	65.25 ± 4.40	56	56	60	62	64	68	72	72
9	207	66.74 ± 5.90	58	60	60	62	68	72	74	75
10	96	68.16 ± 5.61	60	60	60	64	70	72	74	75
11	209	70.52 ± 8.68	60	60	60	66	70	78	80	86
12	320	71 ± 8.32	60	60	60	66	70	80	80	86
13	450	72.20 ± 8.29	60	60	60	70	70	80	80	90
14	439	73.30 ± 9.78	60	60	60	70	70	80	90	90
15	380	74.52 ± 8.36	60	60	60	70	70	80	90	90
16	166	74.40 ± 9.79	60	60	60	70	70	80	90	90
17	86	75.02 ± 9.19	60	60	60	70	70	80	90	90

**Table 5:** Pearson's correlation coefficients

Variables	Systolic BP	Diastolic BP
Height	0.39*	0.31*
Weight	0.39*	0.28*
Body Mass Index	0.16*	0.14*
Age	0.45*	0.38*

* Correlation is significant at the 0.01 level (two tailed), BP: Blood pressure

## DISCUSSION

Hypertension is a major risk factor for cardiovascular and cerebrovascular diseases. Studies indicate that BP increases with age.[12–16] Population-based epidemiological studies show that primary HT is more common among apparently healthy children.[17] Although the prevalence of HT is far less in children than in adults,[18,19] there is enough evidence to suggest that the roots of essential HT extend into childhood.[19–23] The level of ‘normal’ BP varies in different studies due to number of variables such as the size of the rubber bladder within the cotton cuff, type of sphygmomanometer, arm position, whether the fourth or the fifth phase of Korotkoff's sound is used to obtain the DBP, and place and time of BP measurement.[20–21] The differences in patterns of increase in BP between males and females are probably related to certain biological and psychosocial factors. The appearance of secondary sex characters together with the menarche is associated with a high level of anxiety resulting in higher SBP values in girls. However, there are no appreciable differences in the level of the BP of children, aged 5–14 years, between the two sexes.[18–21]

In the present study, the SBP and DBP showed a positive correlation with age, height, weight, and BMI which is consistent with the previously reported studies on BP in children.[19–22] In our study, a significant correlation of height was found with SBP as well as DBP, whereas Sarin *et al*,[22] reported a significant correlation between BP and weight. The boys and girls showed an average annual increase of 2 mm Hg in SBP and 1 mm Hg in DBP in this study which was similar to the finding reported by Sharma *et al*.[23] The relationship between body size and blood pressure has been observed and reported by various authors.[24–25] The age-related increase in BP may be attributable in part to increase in body mass. In the present study, increasing height and weight had a significant positive relationship with SBP and DBP. Voors *et al*,[25] reported that BP correlates more closely to height and body mass than age. A trend of increase in SBP and DBP with age in the present study was observed in both sexes. An increase in SBP and DBP with age has also been reported in Indian children by other authors.[26–29] Gupta *et al*,[16] observed a spurt in SBP between 13–15 years in both sexes. The spurt in SBP between 13–15 years are mainly related to certain biological and psychosocial factors, and puberty timing.[30,31]

The study of childhood HT is significant for several reasons: a) sequelae of long-term HT are irreversible and associated with significant morbidity and mortality, b) childhood BP is the best predictor of adult BP, and c) helps in planning primordial preventive strategies.[32–35] The prevalence of HT in children has been reported to vary between 0.41% to 11.7%.[8,16,26] Previous study from our area[22] suggested the prevalence of HT was 0.96%, whereas in our study it was found to be 5.75%. The reason for low prevalence of HT in previous study[22] may be because of use of means and standard deviation for HT assessment rather than using the more acceptable criterion of 95^th^ percentile of BP values. According to Chadha *et al*,[8] the prevalence of HT in school children is 11.7%. He studied the urban school children where dietary habits, lack of physical activity, and peer pressures could have contributed to such a high incidence. Similarly, Anjana *et al*,[10] concluded that the prevalence of HT is 8.33% and 6.52% among boys and girls, respectively. Previously, it was thought that the prevalence of HT in children in rural India would be less given their life style. However, the present study showed a reasonably high prevalence of 5.75%. This is probably due to rapid urbanization of rural India which has altered the dietary habits, level of physical activity, and social pressures of life.

## CONCLUSION

The patterns of increase in SBP and DBP values were different between males and females and among the different age groups. Age, height, weight, and BMI were positively correlated with both SBP and DBP. The prevalence of HT was found to be higher as compared to the previous report from our own area. It is therefore necessary to check the BP regularly to find out the hidden cases of HT in children including those from the rural areas.
